# 544. Standardizing Competency-Based Onboarding for APP's in Burn and Reconstructive Care: Enhancing Integration, Quality, and Retention

**DOI:** 10.1093/jbcr/irag033.241

**Published:** 2026-04-06

**Authors:** Courtney Denton, Mack D Drake, Hali Wolf, Peter Yen

**Affiliations:** Burn and Reconstructive Center at Chippenham Hospital, Richmond, VA; Burn and Reconstructive Centers of Virginia Chippenham, Richmond, VA; Burn and Reconstructive Centers of Virginia Chippenham, Richmond, VA; Joseph M. Still/ Burn and Reconstructive Centers of America, Birmingham, AL

## Abstract

**Introduction:**

Advanced Practice Providers (APPs) are integral to burn and reconstructive care. However, onboarding practices often vary widely, resulting in inconsistent care delivery and challenges with APP retention. Following provisional burn center state designation in 2022 and full re-designation in 2023, patient volume and acuity increased, prompting the need for a structured onboarding to support workforce growth and retention. We sought to implement a standardized, competency-based onboarding and training model for APPs in burn and reconstructive care aimed at improving clinical readiness, integration, and retention.

**Methods:**

A 12-week modular onboarding curriculum was developed based on national standards (AAPA, AANP) and institutional requirements. The program encompasses six core domains: clinical knowledge, procedural skills, communication, professionalism, inter-professional collaboration, and practice-based learning. APPs rotate through the outpatient clinic, inpatient unit, and operating room with guided evaluations and feedback. Certifications such as Advanced Burn Life Support (ABLS), Hyperbaric Oxygen Therapy (HBOT), and laser scar therapy are offered. Continuous evaluation is provided by physicians, lead APPs, and administrators.

**Results:**

Since implementation in 2022, the onboarding program has significantly enhanced clinical preparedness, team cohesion, and APP confidence. Of the 18 APPs employed over the study period, those who completed the 12-week program (n = 12) showed a 75% retention rate, with an average tenure of 28 months. In contrast, APPs who did not undergo the full program (n = 6) had an average tenure of 14 months and all left the practice. These results exceed national benchmarks, including the 2-year RN retention rate (~62.8%) and annual RN retention (~79.3%) reported by NSI Nursing Solutions, Inc. (2024). The program also aligns with physician retention strategies, such as improved benefits, (Sermo 2023). Additionally, AMN Healthcare (2023) indicates that 36% of nurses consider changing employment within a year highlighting the need for structured onboarding programs.

**Conclusions:**

Our competency-based onboarding has demonstrated effectiveness in improving APP retention and team integration within a high-acuity burn care setting. With retention rates surpassing national averages, this model supports both individual provider development and institutional resilience. These findings suggest the potential for broader application in other acute care environments. As healthcare continues to face workforce retention challenges, structured onboarding remains a promising strategy to promote quality, consistency, and sustainability. Future studies targeting APP-specific cohorts are warranted to further evaluate efficacy and scalability of this retention strategy.

**Applicability of Research to Practice:**

This approach to APP management demonstrates a model to enhance care while improving retention.

**Funding for the study:**

N/A.

